# Thermodynamic Origin of the Vitreous Transition

**DOI:** 10.3390/ma4050869

**Published:** 2011-05-09

**Authors:** Robert Tournier F.

**Affiliations:** Centre National de la Recherche Scientifique, Université Joseph Fourier, Consortium de Recherches pour l’Emergence de Technologies Avancées, B.P. 166, 38042 Grenoble Cedex 09, France; E-Mail: Robert.Tournier@grenoble.cnrs.fr; Tel.: +33-608-716-878; Fax: +33-476-881-280

**Keywords:** Keywords: 64.70 kj glasses, 64.70 P glass transitions, 64.70 pe metallic glasses, 64.70 ph. non metallic glasses, 64.70 pj polymers, 64.60 Q-nucleation

## Abstract

The vitreous transition is characterized by a freezing of atomic degrees of freedom at a temperature T_g_ depending on the heating and cooling rates. A kinetic origin is generally attributed to this phenomenon instead of a thermodynamic one which we develop here. Completed homogeneous nucleation laws reflecting the energy saving due to Fermi energy equalization of nascent crystals and their melt are used. They are applied to bulk metallic glasses and extended to inorganic glasses and polymers. A transition T*_g_ among various T_g_ corresponds to a crystal homogeneous nucleation temperature, leading to a preliminary formation of a cluster distribution during the relaxation time preceding the long steady-state nucleation time of crystals in small samples. The thermally-activated energy barrier ΔG*_2ls_/k_B_T at T*_g_ for homogeneous nucleation is nearly the same in all glass-forming melts and determined by similar values of viscosity and a thermally-activated diffusion barrier from melt to cluster. The glass transition T*_g_ is a material constant and a linear function of the energy saving associated with charge transfers from nascent clusters to the melt. The vitreous transition and the melting temperatures alone are used to predict the free-volume disappearance temperature equal to the Vogel-Fulcher-Tammann temperature of fragile glass-forming melts, in agreement with many viscosity measurements. The reversible thermodynamic vitreous transition is determined by the disappearance temperature T*_g_ of the fully-relaxed enthalpy H_r_ that is not time dependent; the observed specific heat jump at T*_g_ is equal to the proportionality coefficient of H_r_ with (T*_g_ − T_a_) for T ≤ T*_g_ as expected from the enthalpy excess stored by a quenched undercooled melt at the annealing temperature T_a_ and relaxed towards an equilibrium vitreous state. However, the heat flux measurements found in literature over the last 50 years only gave an out-of-equilibrium T_g_ since the enthalpy is continuous at T*_g_ without visible heat jump.

## 1. Introduction

The vitreous state is described, up to now, as a freezing of liquid-state below a temperature T_g_ called vitreous or glass transition, below which the viscosity becomes time dependent with values above 10^12^–10^13^ Pa.s. This transformation at T_g_ is also observed in the heat flow, measured with a technique of differential scanning calorimetry (DSC); endothermic and exothermic heats respectively, depending on the heating and the cooling rates, characterize glass-melt out-of-equilibrium transformations. The glass-forming melt viscosity follows a Vogel-Fulcher-Tammann (VFT) law diverging when the temperature tends to T_0_; T_0_ is much smaller than T_g_ and called the ideal glass transition temperature [1,2,3]. Recent work has shown that the size of heterogeneous regions simultaneously moving to allow a viscous flow grows in the vicinity of the glass transition [4]. The heterogeneous dynamics could also be the result of critical-like fluctuations of static structural order, characterized by a static correlation length diverging towards the ideal glass-transition point T_0_ in the absence of a thermodynamic transition at T_g_. Two glass transition temperatures T_0_ and T_g_ could exist without any connection between the two [5]. In addition, the residual entropy available in undercooled melt, as compared to the crystal one at the glass transition, varies strongly among glasses. The Kauzmann temperature T_k_ has been defined as the temperature at which the crystal fusion entropy would be consumed upon cooling. It could also lead, at thermodynamic equilibrium, to a hidden phase transition following several speculations found in literature [1,2,6].

The high temperature viscosity of some polymer melts, including measurements above the melting point, follows a VFT scaling law giving T_0_ = T_01_ = 0.77 × T_g_ [7]. In the vicinity of the glass transition, the enthalpy relaxation times or the viscosity gives a value T_0_ = T_02_ smaller than T_0l_. Therefore, if T_0_ increases above T_g_ within a narrow range of temperature, it explains why the viscosity values measured at high temperatures do not determine the ideal glass transition. The change of T_0_ occurs around the temperature T_s_ where a break is seen in some volume-*versus*-temperature plots [8]. Many experimental results tend to prove that the ideal glass transition temperature is equal to T_02_ with a viscosity close to T_g_ following a VFT law with T_0_ = T_02_ [3]. The viscosity is an exponential function of B/(T − T_0_) with B nearly proportional to (T_g_ − T_0_) [9].

Following Doolittle’s and Ramachandrarao and Dubey’s works, the free-volume of glass-forming melts would disappear at the ideal glass transition temperature T_0_ [10,11]; then, the two free-volume disappearance temperatures T_0g_ and T_0m_ correspond respectively to our two VFT temperatures T_02_ and T_01_ respectively. Recent numerical simulations of critical-like behavior of glass-forming melts down to T_0_ (assuming that the glass transition only corresponds to a slowing-down of dynamics) suggests that the melt entropy excess at equilibrium must tend to zero for T = T_02_ = T_0g_ instead of T = T_k_, T_k_ being the Kauzmann temperature [5,6,12]. New universal and coherent relations between T_g_, T_0g_ = T_02_ and T_0m_ = T_01_ are proposed in this paper and checked for a series of data in real systems. The associated predictions still need to be clarified.

The vitreous transition, observed by DSC techniques in undercooled melts, is generally time dependent and not strictly reversible, because using the same cooling and heating rates do not lead to the same transformation temperature [13]. These properties underline the kinetic aspects of the transition. A rapid cooling at temperatures far below T_g_ followed by an annealing as a function of time at a temperature T_a_ < T_g_ induces an enthalpy relaxation, saturating to a value H_r_; H_r_ itself tends to zero for the transition temperature T*_g_. From published values of H_r_, it is shown that the reversible transition occurs at T*_g_, and that the transformation into the vitreous state is postponed by quenching the undercooled melt. It is achieved after a time lag equal to the relaxation time by annealing at T = T_a_.

Some DSC techniques are able to separate the specific heat at T*_g_ in two parts; the temperature dependent one is reversible and attached to the thermodynamic aspect of the transition, and the time dependent “irreversible” one is attached to the kinetic aspect. The reversible specific heat jump temperature measured by this technique does not depend on cooling and heating rates and is only a function of the chemical composition [13].

In this paper, we show that crystal homogeneous nucleation occurs at the glass transition T*_g_ of fragile glass-forming melts without any adjustable parameter. This nucleation only depends on the melt composition, and the obtained frozen state is a preliminary step on the long way leading to crystallization. The free energy change associated with a crystal formation in a melt has been accepted for many years, and, regardless of its radius, as if it had the same state equation as a solid outside the melt, which does not reflect the fact that the Fermi energy of a nascent crystal in a metallic melt becomes equal to that of the melt. In order to determine why the vitreous state replaces the crystallized state, a “volume energy saving” ε_v_ has been added to the Gibbs free-energy change associated with crystal homogeneous nucleation in melt, to obtain the equalization of Fermi energies transferring free electrons from the crystal to the melt [14,15,16]. The energy saving ε_v_ is equal to ε_lps_ΔH_m_/V_m,_ ΔH_m_, V_m_, and ε_lps_ respectively being the molar fusion heat, the molar volume, and the energy saving coefficient, where ε_lps_ is a numerical coefficient depending on the reduced temperature θ = (T − T_m_)/T_m_ [16]. The indexes s and l are related to solid and liquid states. The index p is suppressed for unmelted crystals acting as growth nuclei and replaced by the index g for crystals resulting from homogeneous nucleation in glass-forming melts. The value of ε_lps_ can be predicted using the VFT temperatures T_01_ and T_02_ viewed as the disappearance temperatures T_og_ and T_0m_ of fragile-glass-forming melt free-volume and of Fermi energy difference between crystal and melt [14,15]. The vitreous transition T*_g_ only depends on the energy saving coefficient. The experimental values of T_0g_ can also be used to predict the vitreous transition temperature T*_g_.

In this model, the crystal growth starts at the crystal nucleation temperature with a cluster preliminary formation on the long way leading to crystallization. It is locked at the vitreous transition by a freezing without any change of enthalpy and entropy. The same analysis is successfully applied to some polymers and non-metallic glass-formers. The presence of a similar “volume energy saving”, governing the vitreous transition, is determined. This phenomenon is probably due to a free energy which depends on the number of molecules or atoms in a small crystal having a noncritical radius or to an electrostatic interaction between uncompensated average charges proportional to n^1/2^ carried by nascent crystals built from a random distribution of ions on various sub-lattices and screened by ionic charges of the melt.

**Figure 1:**
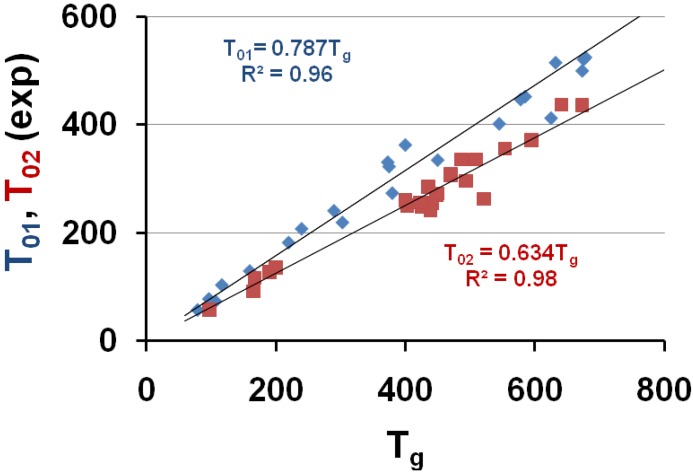
The VFT temperatures T_01_ and T_02_ given in Table 1 are plotted *versus* T_g_; T_02_ corresponds to measurements in the vicinity of T_g_ and T_01_ includes viscosity measurements at higher temperatures. T_01_ ≅ 0.787 T_g_ and T_02_ ≅ 0.634 T_g_.

This paper is built as follows: Section 2. Model; 2.1. New equations governing the crystal nucleation; 2.2. The ideal glass transition T_0_ and the energy saving associated with crystal formation; Section 3. Review of experimental results and discussion; 3.1 Presentation of Table 1 and Figure 1; 3.2. Homogeneous nucleation time of crystals and relaxation time; 3.3. The thermodynamic vitreous transition T*_g_ at the disappearance temperature of the fully-relaxed enthalpy; 3.4. The crystal homogeneous nucleation temperature at T*_g_; 3.5. Volume energy saving associated with nascent crystals in non-metallic glass-forming melts; 3.6. Thermodynamic origin of the relaxed enthalpy and out-of-equilibrium transition temperatures T_g_; Section 4. Summary and complementary information on the two crystal nucleation temperatures; Section 5. Conclusions.

## 2. Model

### 2.1. New Equations Governing the Crystal Nucleation

The classical equation describing the Gibbs free-energy change associated with a crystal formation, predicts the absence of surviving crystals above the melting temperature T_m_ [17]. On the contrary, their existence is predicted far above T_m_ if an energy saving per volume unit ε_v_ = ε_lps_ × ΔH_m_/V_m_ is added. The maximum undercooling ratio θ_1_ = (T_1_ − T_m_)/T_m_ is observed as being of the order of −0.2 in liquid elements using droplet sizes of 50–10,000 micrometers instead of −2/3 [18]. This pseudo-maximum has until now been considered to be the maximum of the homogeneous nucleation rate in contradiction with a detailed study of crystallization temperature of gallium droplets as a function of their diameter. Two undercooling temperature dwells, instead of one, corresponding to θ_1_= −0.28 and θ_1_= −0.5 have been observed for diameters varying from 1 to 1,000 micrometers [19]. This phenomenon is induced by a distribution of surviving crystal radii between two boundary radii after overheating. A large overheating rate has to be applied in order to melt a part of them and obtain an undercooling rate dwell of about −0.2 [20]. The second dwell corresponding to stronger undercooling rates, is due to numerous surviving crystals having the smallest radius [21,22,23]. The sample radius has to be strongly reduced to observe it. The energy saving ε_v_ depends on the Fermi energy difference between solid and liquid and is an even function of the reduced temperature θ = (T − T_m_)/T_m_ as shown in (1) [14,16]:

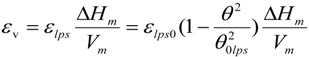
(1)


Consequently, the fusion heat of unmelted crystals remains equal to ΔH_m_ regardless of their radius, because dε_ls_/dθ = 0 and d(ΔG*_2ls_)/dθ = ΔH_m_ × 4πR^3^/3/V_m_ at θ = 0, with ΔG*_2ls_ being the completed Gibbs free energy change for a crystal formation in a melt given by (2):

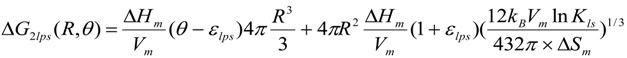
(2)
k_B_ being the Boltzmann constant, ΔS_m_ the fusion entropy per mole and lnK_lps_ given by (12). The volume energy saving ε_v_ is added to the classical Gibbs free energy θ × ΔH_m_/V_m_ and the surface energy is modified by the factor (1 + ε_ls_). The classical equation ΔG_1ls_ (θ) is deduced from (2) with ε_ls_ = 0 [17,18,19]. The experimental values of surface energy and lowest undercooling temperatures of many liquid elements have been used to determine the new surface energy and ε_ls_ in (1,2) [17]. This Equation (2) allows us to calculate the unique homogeneous nucleation temperature θ_2ls_ = −2/3 and the crystallization temperatures of liquid elements from intrinsic growth nuclei without any adjustable parameter [21,22,23].

The energy saving becomes equal to zero at T_m_ above a radius a little larger than the critical radius for crystal growth nucleation to obey, at equilibrium, the classical J. W. Gibbs’s phase coexistence rule and because a free electron cannot be transferred from the crystal to the melt with an energy saving larger than the Fermi energy . The crystal homogeneous nucleation maximum-rate temperature T_2lps_ (or θ_2lps_) defined by (3) in the undercooled melt is respectively called θ_2lgs_ or θ_2ls_ with values of ε_lps0_ equal to ε_lgs0_ or ε_ls0_:

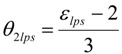
(3)


The θ_2lps_ only depends on the coefficient ε_lps_ defined by (1) and (3) and does not depend on other material properties. It is equal to (T_olps_ − T_m_)/T_m_ where T_olps_ is equal to T_0m_ or T_0g_. The critical energy barrier, the critical radius and θ_2lps_ given by (3) have been calculated assuming that ε_lps_ is not radius dependent. This assumption works because the influence of dε_lps_/dR is negligible on the critical parameters of a lot of melts. Equations (1–3) have already been used to predict the time-temperature-transformation diagrams of Mg_65_Y_10_Cu_25,_ Zr_41.2_Ti_13.8_Cu_12.5_Ni_10_Be_22.5_ and Pd_43_Cu_27_Ni_10_P_20_ melts [14,15] and the undercooling temperature dwells of liquid elements, in agreement with the experiments without using any adjustable parameter [21,22,23]. Unequal coefficients ε_ls_ ≠ ε_lgs_ would lead to θ_2ls_ ≠ θ_2lgs_. Equal coefficients ε_lps_ = ε_ls_ = ε_lgs_ would lead to the same homogeneous nucleation temperature; the glass transition would be equal to the crystallization temperature.

### 2.2. The Ideal Glass Transition Temperature T_0_ and The Energy Saving Associated with Crystal Formation

Many experiments show the presence of numerous intrinsic growth nuclei in melts. Glasses can give rise to about 10^25^ nanocrystals per m^3^ within a few hours when they are annealed above the vitreous transition. This number is much too large to be compared with the classical homogeneous nucleation rate. High resolution microscopy reveals the existence of “mean range order” clusters called MRO with a radius of about one nanometer in amorphous Fe_83_B_17_ [24]. These entities are not viewed as surviving crystals because they do not exist in the literature. They are as numerous as the nanocrystals and could be growth nuclei. A recent observation of an irreversible viscosity of Fe_85_B_15_ far above the liquidus temperature could also be a sign of the existence of surviving crystals up to temperatures as high as 1.3 T_m_ [25].

A liquid-solid transition is accompanied by Fermi energy and volume changes in metallic glass-forming melts. The free volume change ΔV and the Fermi energy difference ΔE_F_ are expected to disappear at the same reduced temperature θ_0lps_ > −2/3 and to be maximum at the melting temperature (θ = 0) in agreement with the thermal variation given by (1) of the energy saving coefficient ε_lps_ of liquid elements between θ_0lps_ = θ_2lps_ ≅ −2/3 and θ = 0 [14,15,16].

The glass-forming melt viscosity is represented by a VFT relation given by (4) depending on three parameters η_0_, B and T_0_, in the region of the glass transition T_g_ [1,2,3]. Its variation by several orders of magnitude above and close to T_g_ is used to determine T_0_ [3,26] because including viscosity values measured far above the melting temperature increases the T_0_ value [27,28]; it is important to fix η_0_ ≅ N_A_ × h/V_m_ with N_A_ Avogadro’s number, h Planck’s constant and V_m_ the molar volume to evaluate T_0_ and B in (4) [28]:

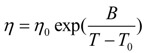
(4)
The relaxation time dependence in the temperature range between the onset and the end of the endothermic transition is observed by DSC. At a constant heating rate, the relaxation time τ is also described by the VFT-type relation (5):

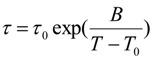
(5)
T_0_ and B are determined using a pre-exponential coefficient τ_0_ of about 10^−14^ s [3,27,28].

The free-volume of glass-forming melts is a linear function of (T−T_0_). Doolittle’s relation introduces the free volume in the exponential of (4,5) [10]; ΔV would be equal to zero for T = T_0_ in the absence of vitreous transition. The values of T_0_ would correspond to the extrapolated free-volume disappearance temperature. Some measurements exist [29,30]. For example, the Pd_43_Cu_27_Ni_10_P_20_ volume in the liquid and solid states are known down to an extrapolated value ΔV = 0 occurring for T_02_ ≅ 452 K. In Pd_40_Cu_30_Ni_10_P_20_, the VFT law leads to T_02_ = 447 K [31].

The minimum value of ε_lps0_ in undercooled melt can be calculated only knowing T_01_ (or θ_01_) and T_02_ (or θ_02_) from VFT equations [14,15]. The quadratic equation (6) is obtained applying (1) and (3) for θ = θ_2lps_:

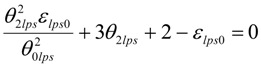
(6)


There are two solutions for θ_2__lps_ when ε_lps0_ is larger than a minimum value as already described [14,15]. The relations (7) and (8) between ε_lps0_, θ_2lps_ and 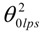 are respected when the double solution corresponds to a minimum value of ε_lps0_ larger than 1. It is given by (7,8) and occurs when 9 − 4 × (2 − ε_lps0_) × ε_lps0_/θ^2^_0lps_ = 0:

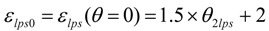
(7)

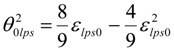
(8)


The knowledge of θ_02_ and θ_01_ from VFT equations chosen respectively equal to θ_0lgs_ and θ_0ls_ and the use of (7) or (8) determine ε_lps0_ and θ_2lps_ and the minimum value of ε_lps0_. The existence of two glass-former classes and their boundaries are predicted completing Angel’s description, if we assume that θ_2lgs_ = θ*_g_ = (T*_g_ − T_m_)/T_m_ [3].

Fragile bulk glasses correspond to θ_0lgs_ = θ_0g_ > −2/3 and ε_lgs0_ > 1, and fragile and quenched glasses to ε_lgs0_ < 1 and θ_2lgs_ = θ_0g_ = −2/3. The undercooled liquid state can be recovered by heating the glass above T_2lgs_ (or θ_2lgs_) because the condition ε_lgs0_ > 1 stabilizes it. It is not recovered when ε_lgs0_ < 1 and θ_0g_ = −2/3 because there is no minimum value of ε_lgs0_. All predictions of (7,8) are related to the free-volume disappearance temperature of fragile undercooled melts which is equal to the ideal glass transition temperature. Angel’s classification does not fix a quantified boundary between strong and fragile ones.

The strong glass-forming melts correspond to θ_0lgs_ ≤ −2/3, ε_lgs0_ < 2, and θ_2lgs_ > −2/3. They also have a viscosity larger than fragile melts with temperature dependence close to Arrhenius law. Their vitreous transition temperature can be a very large fraction of the melting temperature. The largest value of ε_lps0_ is deduced from the experimental values of θ_0lps_ and θ_2lps_ using (6). Strong glass-forming melts have a homogeneous nucleation temperature always larger than T_m_/3 without metastable values of ε_lps0_ regardless of the energy saving.

## 3. Review of Experimental Results and Discussion

### 3.1. Presentation of Table 1 and Figure 1

The melting temperatures T_m_, the experimental glass transition temperatures T_g_ or θ_g_ = (T_g_ − T_m_)/T_m_, the VFT temperatures T_01_ determined up to temperatures much higher than T_g_, the VFT temperatures T_02_ determined by viscosity or relaxation time measurements in the vicinity of T_m_, the free-volume disappearance temperatures T_0g_ = T_0lgs_ calculated using (7,8) and (18) considering that T_g_ is, in a first approximation, nearly equal to the thermodynamic glass nucleation temperature T*_g_, the saving energy coefficients ε_ls0_(θ = 0) of crystals surviving in the melt far above T_g_ calculated using (19), the saving energy coefficients ε_lgs0_ (θ = 0) of nascent crystals homogeneously nucleated in the melt near T_g_ calculated using (18), the free-volume disappearance temperatures T_0m_ = T_01_ calculated using (7,8,19,20), T_g_, and the references [32,33,34,35,36,37,38,39,40,41,42,43,44,45,46,47,48,49,50,51,52,53,54,55,56,57,58,59,60,61,62,63] are given in Table 1.

Properties of 20 non-metallic glasses and polymers are numbered with references. B_2_O_3_ is numbered 11 and 12. Two values of T_g_ are used. This glass is not easily crystallized. It gives rise to two crystallographic structures and its highest melting temperature is 783 K. SiO_2_ N°3 is a strong glass (θ_0l_ < −0.666). Hevea rubber N°50 is just at the limit separating strong glasses from fragile ones because T_og_ is a little larger than T_m_/3.

Properties of 28 bulk metallic glasses are used and numbered with references. The difference of liquidus and solidus temperatures is sometimes too large. A homogeneous melt has a well-defined Fermi energy. The melting temperature has been chosen between these two limits looking at the DSC profile. Two melting temperatures T_m_ = 728 K and T_l_ = 925 K are used for La_55_Al_25_Ni_20_ N° 21 and 22. The first one corresponds to the largest endothermic peak and the second one to the liquidus. We find ε_ls0_ = 1.65 and about 1.51 respectively. Two melting temperatures are also used for Pd_40_Ni_40_P_20_ N°24 and 25, T_m_ = 987 K and T_m_ = 884 K leading to ε_ls0_ = 1.63 and 1.56 respectively. The melting temperatures of La_55_Al_25_Ni_5_Cu_15_ N°28, La_55_Al_25_Ni_15_Cu_5_ N°32 and La_55_Al_25_Ni_5_Cu_10_Co_5_ N°41 are respectively chosen equal to 700 K instead of 878 K, 729 K instead of 899 K and 754 K instead of 822.5 K.

Experimentalists call all glasses characterized by a crystallization temperature T_x_ occurring near T_g_ “conventional”. Among them, glasses have a value of T_g_ close to T_m_/2. The glass transitions of Al_87_Co_4_Ce_9_, Al_87_Co_6_Ce_7_, Al_87_Co_8_Ce_5_, Al_85_Co_10_Ce_5_ and Al_90_Co_5_Ce_5_ are not reported because they cannot be distinguished from the crystallization temperature T_x_, in the absence of endothermic heat before crystallization in a DSC run [62]. The values of θ_x_ = (T_x_ − T_m_)/T_m_) are respectively equal to −0.481, −0.523, −0.533, −0.543, −0.567. Au_0.77_Ge_0.136_Si_0.094_ N°51 is characterized by a crystallization temperature T_x_ occurring above and very close to T_g_ with θ_g_ = −0.539 [63]. All these alloys have an energy saving coefficient larger than 1. They could belong to the fragile glass class because they have a VFT temperature T_0_ larger than T_m_/3.

**Table 1:** Some properties of 46 glass-forming melts are presented: T_m_ the melting temperature; T_g_ the vitreous transition temperature, θ_g_ = (T_g_ − T_m_)/T_m_, T_01_ and T_02_ the Vogel-Fulcher-Tammann temperatures, as found in various references; T_0g_ the free-volume disappearance temperature calculated from T_g_ and not from T*_g_; ε_ls0_ the energy saving coefficient of tiny crystals surviving in the melt and acting as growth nuclei; ε_lgs0_ the energy saving coefficient of homogeneously-nucleated crystals in the melt; ε_ls0_ and ε_lgs0_ being used to calculate T_0m_ and T_0g_; T_0m_ the free-volume disappearance temperature also calculated from T_g_ and not from T*_g_; and references.

	Glass	T_m_	T_g_	θ_g_	T_01_	T_02_	T_0g_	ε_ls0_	ε_lgs0_	T_0m_	References
1	As_2_S_3_	585	481	−0.178		270	319	1.822	1.732	363	[32,33]
2	Propylene glycol	214	167	−0.220		117	108	1.780	1.671	125	[26,34,35]
3	SiO_2_	1,993	1,473	−0.261		300			1.36		[2,36]
4	Propylene carbonate	217	160	−0.263	130		102	1.737	1.606	119	[35]
5	polystyrene	513	375	−0.269	323		239	1.731	1.596	280	[26,37]
6	Pd_43_Ni_10_Cu_27_P_20_	802	585	−0.271	452		372	1.729	1.594	436	[29,38,39]
7	O-Terphenil	329	240	−0.271	208		153	1.729	1.593	179	[3,26,40]
8	Pd_40_Cu_30_Ni_10_P_20_	823	578	−0.298	447		366	1.702	1.553	432	[29,31,39]
9	Salol	315	220	−0.302	183		139	1.698	1.548	165	[3,26,35]
10	As_2_Se_3_	645	450	−0.302	335		285	1.698	1.547	337	[41]
11	B_2_O_3_	783	545	−0.304	402		345	1.696	1.544	408	[3,11,32]
12	B_2_O_3_	783	521	−0.335		263	330	1.665	1.498	393	[3,11,32]
13	Bromopentane	158	107	−0.323	74		68	1.677	1.516	80	[26]
14	ZnCl_2_	565	380	−0.327	274		241	1.673	1.509	286	[2,26]
15	Butene 1	88	59	−0.330			37	1.670	1.506	44	[2]
16	Zr_41.2_Ti_13.8_Cu_12.5_Ni_10_Be_22.5_	937	625	−0.333	413		396	1.667	1.501	472	[39,42]
17	La_55_Al_25_Ni_10_Cu_10_	662	441	−0.334		255	280	1.666	1.499	333	[43,44,45]
18	2 Methylpentane	120	80	−0.338	58		50	1.663	1.494	60	[2,26,46]
19	Toluene	178	117	−0.343	104		74	1.657	1.485	89	[47]
20	Glycerol	293	190	−0.352		128	121	1.648	1.473	144	[26,34,35]
21	La_55_Al_25_Ni_20_	728	470	−0.354		307	299	1.646	1.469	358	[43,44,45]
22	La_55_Al_25_Ni_20_	925	470	−0.492		309	330	1.508	1.262	394	[43,44,45]
23	PET = (C_10_H_8_O_4_)n	542	342	−0.369			219	1.631	1.446	262	[2]
24	Pd_40_Ni_40_P_20_	884	554	−0.373		356	355	1.627	1.440	425	[48,49]
25	Pd_40_Ni_40_P_20_	987	554	−0.439		356	369	1.561	1.342	442	[48,49]
26	Pt_57.5_Cu_14.7_Ni_5.3_P_22.5_	813	509	−0.374		336	326	1.626	1.439	390	[50]
27	Pd_0.775_Cu_0.06_Si_0.165_	1,015	632	−0.377	515		405	1.623	1.434	486	[51]
28	La_55_Al_25_Ni_5_Cu_15_	700	436	−0.378		286	279	1.622	1.433	335	[43,44,45]
29	Zr_52.5_Cu_17.9_Ni_14.6_Al_10_Ti_5_	1,090	675	−0.381	521		434	1.619	1.429	519	[52]
30	Pt_57.3_Cu_14.6_Ni_5.3_P_22.8_	788	487	−0.382		336	313	1.618	1.427	375	[50]
31	Se	491	303	−0.383	220		195	1.617	1.426	233	[2,53]
32	La_55_Al_25_Ni_15_Cu_5_	729	449	−0.384		273	289	1.616	1.424	346	[43,44,45]
33	Au_49_Ag_5.5_Pd_2.3_Cu_26.9_Si_16.3_	655	403	−0.385		250	259	1.615	1.423	311	[39]
34	Ethanol	159	97	−0.390	78	58	63	1.610	1.415	75	[2,11,32]
35	Zr_58.5_Cu_15.6_Ni_12.8_Al_10.3_Nb_2.8_	1,110	673	−0.394		437	435	1.606	1.409	522	[54]
36	Zr_57_Cu_15.4_Ni_12.6_Al_10.3_Nb_5_	1,120	678	−0.395	525		438	1.605	1.408	526	[52]
37	Pr_55_Ni_25_Al_20_	820	494	−0.398		296	320	1.602	1.404	384	[55]
38	Ti_41.5_Cu_37.5_Ni_7.5_Zr_2.5_Hf_5_Sn_5_Si_1_	1,176	693	−0.411			452	1.589	1.384	543	[56]
39	Cu_47_Ti_34_Zr_11_Ni_8_	1,144	673	−0.412	500		439	1.588	1.382	527	[57]
40	Y_56_Al_24_Co_20_	1,085	636	−0.414	614		416	1.586	1.379	499	[58]
41	La_55_Al_25_Ni_5_Cu_10_Co_5_	754	439	−0.418		241	288	1.582	1.374	345	[43,44,45]
42	Mg_59.5_Cu_22.9_Ag_6.6_Gd_11_	734	425	−0.421		249	279	1.579	1.369	335	[59]
43	Mg_61_Cu_28_Gd_11_	737	422	−0.427		256	278	1.573	1.359	334	[59]
44	Mg_65_Cu_25_Y_10_	739	400	−0.459	363	260	271	1.541	1.312	325	[27]
45	Zr_46.75_Ti_8.25_Cu_7.5_Ni_10_Be_27.5_	1,070	595	−0.444		372	398	1.556	1.334	477	[60]
46	Cyclo-octanol	298	165	−0.446		92	110	1.554	1.331	133	[26]
47	Zr_65_Al_10_Ni_10_Cu_15_	1,161	641	−0.448		437	430	1.552	1.328	516	[39]
48	Ce_60_Al_10_Ni_10_Cu_20_	677	373	−0.449	331		250	1.551	1.326	300	[61]
49	Al_87_Co_4_Ce_9_	1,104	573	−0.481			397	1.519	1.279	475	[62]
50	Hevea rubber	421	200	−0.525		136	147	1.475	1.213	174	[32]
51	Au_0.77_Ge_0.136_Si_0.094_	629	290	−0.539	241		217	1.461	1.192	257	[63]

The temperatures T_01_ and T_02_ are plotted as a function of the vitreous transition temperature T_g_ in Figure 1; the upper straight line uses the equation T_01_ = 0.787 × T_g_ and corresponds to a similar law T_01_ = 0.77 × T_g_ already observed for 7 other polymers [7,64]; the lower straight line uses the equation T_02_ = 0.634 × T_g_.

### 3.2.Homogeneous Nucleation Temperature of Vitreous Phase and Relaxation Time

The calculation of the crystal nucleation full time t includes not only the steady-state nucleation time t_sn_ defined by v × t_sn_ = 1 in (10) at the nucleation temperature, but also the time lag τ^ns^ in crystal transient nucleation defined by (9) [2] with K_lgs_ defined by (12):

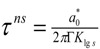
(9)

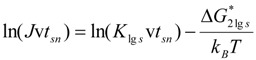
(10)

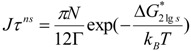
(11)

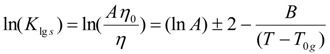
(12)


The steady-state nucleation rate is J, the volume sample v, Zeldovitch’s factor Γ, the critical energy barrier ΔG*_2lgs_/k_B_T, a*_0_ = π^2^/6, the atom or molecule number per volume unit N. The quantity lnK_ls_ is equal to lnA ≅ 90 ± 2 for liquid elements in a broad temperature scale; lnA is a little smaller for crsystallization of glass-forming melts. In the vicinity of T*_g_, and assuming that T*_g_ (or θ*_g_) is equal to a crystal homogeneous nucleation temperature T_2lgs_ (or θ_2lgs_), when J = 1, the crystal transient nucleation time τ^ns^ viewed as the relaxation time, can be calculated with (15) using the critical parameters (13) and (14):

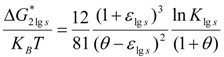
(13)

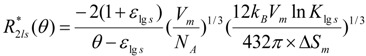
(14)

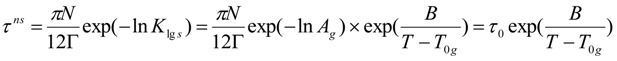
(15)


The coefficient of lnK_lgs_ in (13) is equal to 1 for θ = θ_2lgs_ = (ε_ls_ − 2)/3 when the crystal-steady-state nucleation time is minimum. The A_g_ is the A value defined by (12) at the glass transition, which is used for the homogeneously-nucleated cluster formation. Zeldovitch’s factor given by (16) is calculated below, as a function of the number J_c_ of molecules or atoms in a spherical crystal of critical radius R*_2lgs_, given by (14) at θ_2lgs_ = θ∗_g_. The pre-exponential time τ_0_ given by (17) depends on A_g_ and ln(τ^ns^/τ_0_) is equal to B/(T*_g_ − T_0g_) ≅ 36.5 − 39 at T*_g_ [3,9]:

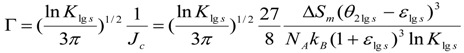
(16)

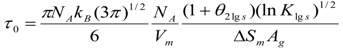
(17)
where ΔS_m_ is the fusion entropy per mole, N_A_ the Avogadro number, θ_2lgs_ defined by (3), N_A_k_B_ = 8.32 Joule. The temperature T_2lgs_ (or θ_2lgs_) is a constant of the material and a unique function of the energy saving ε_lgs_ in (3). It does not strictly depend on viscosity. Nevertheless, the viscosity has a strong influence on the occurrence of the maximum nucleation rate temperature because the critical energy barrier ΔG*_2lgs_/k_B_T is proportional to lnK_lgs_ and the numerical coefficient of lnK_lgs_ in ΔG*_2lgs_/k_B_T is equal to about 1 in a broad window of temperatures above T*_g_. The critical energy barrier ΔG*_2lgs_/k_B_T is nearly the same at T*_g_ for all glass-forming melts and lnK_lgs_ decreases with the increase in viscosity. The nucleation rate is at a maximum when lnK_lgs_ becomes exactly equal to ΔG*_2lgs_/k_B_T_g_. This event occurs at T*_g_ (or θ*_g_) = T_2lgs_ (or θ_2ls_) for similar viscosity values because τ_0_, and the relaxation time 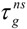 are nearly the same in all glass-forming melts at T_g_.

The relaxation time 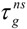 is generally of the order of 100 s at T_g_. A value τ_0_ = 1.4 × 10^−14^ s is deduced with B/(T_g_ − T_0g_) = 36.5 and τ_0_ = 3.14 × 10^−15^ s with B/(T_g_ − T_0g_) = 38 in all glasses [3,9,27]. Equation (17) giving τ_0_ is used to determine lnA_g_ only, depending on {ln(1 + θ_2lgs_] − ln[V_m_ × ΔS_m_].

With τ_0_ = 1.4 × 10^−14^ s, the lnA_g_ is equal to 100.7 in N°6 Pd_43_Ni_10_Cu_27_P_20_ (V_m_ = 8 × 10^−6^ m^3^, ΔS_m_ = 8.74 J/K, θ_2lgs_ = −0.271) and to 96.4 in N°20 Glycerol (V_m_ =73.07 × 10^−6^ m3, ΔS_m_ = 62.42 J/K, θ_2lgs_ = −0.352).

With τ_0_ = 3.14 × 10^−15^ s, the lnA_g_ is respectively equal to 102.2 and 97.9 for the same liquids with an increase of the product V_m_ × ΔS_m_ by a factor 66.

With τ_0_ = 1.4 × 10^−14^ s, the average value of lnA_g_ is 98.5 ± 2 and with τ_0_ = 3.14 × 10^−15^ s, lnA_g_ = 100 ± 2. The lnK_lgs_ and the thermally-activated energy barrier ΔG*_2lgs_/k_B_T_2_ are always equal to 62 ± 2 in all glass-forming melts at T = T_g_.

Equation (15) shows that the assumption of a value of τ_0_ being the same in all melts can be replaced by a nearly-constant value of A_g_; lnA_g_ is about 15% larger than the one found for crystal nucleation from surviving crystals in several glass-forming melts at higher temperatures [14]. Then, the time lag of a transient nucleation to produce a crystal nuclei distribution, built from surviving crystals and ready for steady-state nucleation, is about 10^6^ times larger than the time lag required for a homogeneously-nucleated cluster distribution formation. The steady-state nucleation time t_sn_ is, in addition, equal to 10^9^ s for v = 10^−9^ m^3^. A nucleus distribution with cluster size close to the critical value is created just near T_g_ when the relaxation time is minimum. In Turnbull and Fisher’s model, lnK_ls_ is nearly equal to ln(N_A_k_B_T*_g_/V_m_h) − Δf*/k_B_T*_g_ where h is Planck’s constant and Δf*/k_B_T*_g_ is a thermally-activated energy barrier for atom diffusion from the melt to the homogeneously-nucleated cluster which is smaller than the one from the melt to a surviving crystal [65]. This weakening could be due to formation of clusters containing many vacancies on their various sub-lattices during homogeneous nucleation. Surviving crystals are expected to be well-crystallized because they are part of previously-crystallized materials. They did not melt above T_m_ and they are very stable with their fusion heat equal to the bulk one [21,22,23].

### 3.3. The Thermodynamic Vitreous Transition T*_g_ at the Disappearance Temperature of the Fully-Relaxed Enthalpy

**Figure 2:**
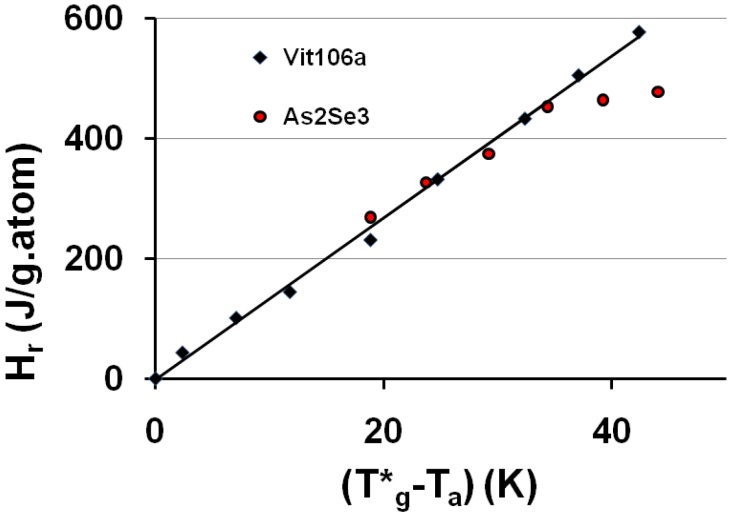
The saturated value of the relaxed enthalpy H_r_ is plotted *versus* (T*_g_ − T_a_), T*_g_ being the thermodynamic vitreous transition and T_a_ the annealing temperature; for N°35 vit 106a (Zr_58.5_Cu_15.6_Ni_12.8_Al_10.3_Nb_2.8_), T*_g_ = 690 K and the slope of the straight line ΔC_pgl_ = 13.5 J/g.atom and for N° 10 As_2_Se_3_, T*_g_ = 462 K and ΔC_pgl_ = 13.5 J/g.atom [54,66,67]. The corresponding values of T_0g_ calculated from T*_g_ are 443 K and 293 K. The deviation from the straight line is due to the approach of the Kauzmann temperature of As_2_Se_3_ [67].

**Figure 3:**
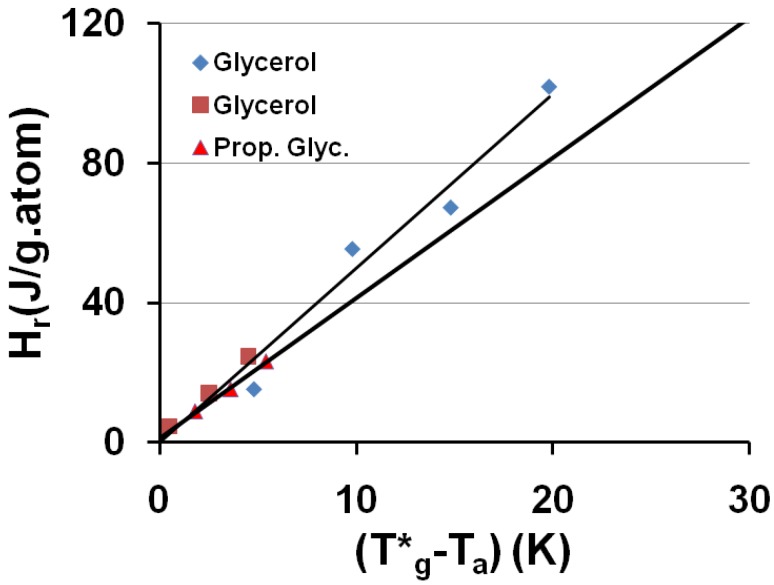
The saturated value of the relaxed enthalpy H_r_ is plotted *versus* (T*_g_ − T_a_), T*_g_ being the thermodynamic vitreous transition and T_a_ the annealing temperature; for N° 20 glycerol, T*_g_ = 189.8 K and the slope of the straight line ΔC_pgl_ = 4.99 J/g.atom and for N° 2 propylene glycol T*_g_ = 171 K and ΔC_pgl_ = 4.02 J/g.atom. The corresponding values of T_0g_ calculated from T*_g_ are 121 and 112 K respectively. The largest values of H_r_ are due to [68] and the smallest ones to [69].

A relaxed enthalpy is measured by DSC, after quenching the undercooled liquid to much lower temperatures than T_g_ and annealing it at a temperature T_a_ smaller than T_g_, during the relaxation time necessary to obtain its maximum value H_r_.. The structural relaxation is viewed as a transformation of the quenched undercooled liquid state in a fully-frozen state. This exothermic heat varies linearly with (T*_g_ − T_a_) as shown in Figure 2 and Figure 3. The fully-relaxed enthalpies H_r_ of As_2_Se_3_ and Zr_58.5_Cu_15.6_Ni_12.8_Al_10.3_Nb_2.8_ (vit106a) are plotted in Figure 2 using values of T*_g_ equal to 462 and 690 K instead of T_g_ = 450 and 673 K respectively [54,66,67]. The calculated temperatures T_0g_ are respectively 293 K and 437 K as compared to T_02_ = 335 and 437 K. The vitreous transition T*_g_ of As_2_Se_3_ exactly corresponds to the mid-point of the reversible specific heat jump [67]. In Figure 3 we describe the relaxed enthalpy variation of propylene glycol and glycerol extracted from two different publications with T*_g_ = 171 and 189.8 K instead of T_g_ = 167 and 190 K respectively [68,69]. The calculated temperatures T_0g_ are respectively 112 K and 121 K as compared to T_02_ = 117 and 128 K.

The specific heat excess of the undercooled melt ΔC_pgl_ can be directly calculated from dH_r_/dT_a_ = ΔC_pgl_ because H_r_ is a linear function of T_a_ and 
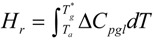
. The following calculated values of ΔC_pgl_ are in good agreement with the specific heat difference ΔC_pls_ between solid and liquid; for propylene glycol, ΔC_pgl_ = 52.3 and ΔC_pls_ = 67.3 J/mole/K [68,69]; for glycerol, ΔC_pgl_ = 69.9 and ΔC_pls_ ≅ 79.4 J/mole/K [32,68,69]; for vit106a, ΔC_pgl_ = 13.5 and ΔC_pls_ ≅ 15.5 J/mole/K [54]; for As_2_Se_3_, ΔC_pgl_ = 67 J/mole/K from the relaxed enthalpy and 67 J/mole/K from the reversible specific heat [67]. The specific heat jump ΔC_pls_ is a little too large at T_g_ when it is measured at a too low heating rate because it still contains an endothermic contribution except when stepscan techniques are used [13].

The specific heat excess of an undercooled melt tends to zero at the Kauzmann temperature as shown by the fact that the derivative dΔH_r_/dT of As_2_Se_3_ tends to zero at this temperature as reproduced in Figure 2 [66,67]; the Kauzmann temperature T_k_ is an actual temperature of undercooled melts instead of a virtual one [67].

### 3.4. The Crystal Homogeneous Nucleation Temperature at T*_g_

The vitreous transition T*_g_ (or θ*_g_) is viewed as occurring at the crystal steady–state nucleation maximum-rate temperature T_2lgs_ (or θ_2lgs_). In this case, the glass transition being a material constant has to obey (6). The energy saving approximate coefficients ε_lgs0_ in Table 1 are given by (18) using T_g_ which is not known instead of T*_g_:

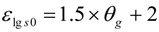
(18)


**Figure 4:**
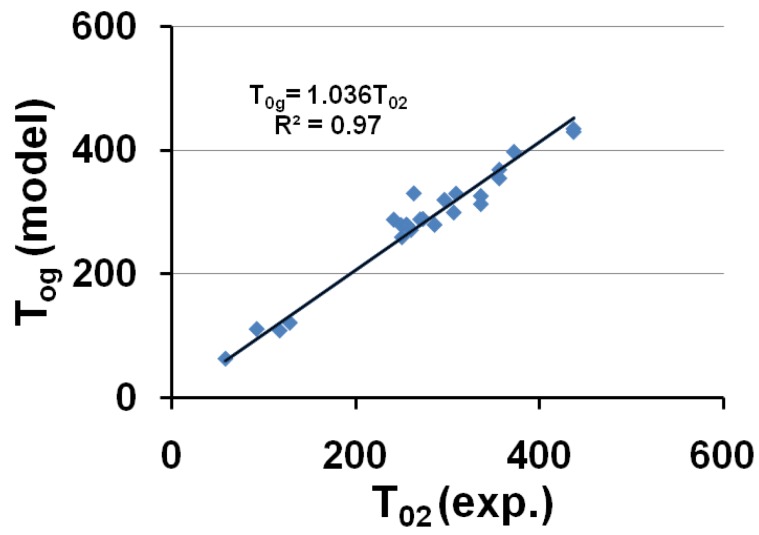
The calculated values of the free-volume disappearance temperature T_0g_ of fragile glass-forming melts are plotted *versus* the VFT temperatures T_02_ determined by measurements in the vicinity of T_g_; T_0g_ ≅ 1.036 T_02_.

The corresponding temperatures T_0g_ = T_0lgs_ (or θ_0g_ = θ_0lgs_) listed in Table 1, are calculated from ε_lgs0_ determined by (18) and (7,8). The calculated temperature T_0g_ of the free volume disappearance is plotted as a function of the VFT temperature T_02_ in Figure 4. The average of T_0g_ is 3.6% larger than that of T_02_. These quantities are nearly equal if we consider that T_g_ is an out-of-equilibrium temperature which is not exactly equal to the thermodynamic transition T*_g_. The model works and is able to predict the VFT temperature of fragile glass-forming melts when T*_g_ is known.

The energy saving coefficients ε_lgs0_ and ε_ls0_ are not equal and correspond to the two reduced temperatures θ_0g_ and θ_0m_ given by (6). The coefficient ε_ls0_ is calculated using T_0m_ and T_g_ of N° 29 vit105 (Zr_52.5_Cu_17.9_Ni_14.6_Al_10_Ti_5_) because the scaling law T_0m_ = 0.77 × T_g_ [7,64] is obeyed by this material. Δε (θ_g_) = (ε_ls0_ − ε_lg0_) is equal to 0 for θ_g_ = 0 and 0.19 for θ_g_ = −0.381. These two particular values are used to determine a possible scaling law followed by Δε. The crystal nucleation maximum-rate temperature θ_2ls_ is given by (7,8) as a function of ε_ls0_; then, ε_ls0_ and ε_ls0_ − ε_lg0_ = Δε have to be linear functions of θ_g_ given by (11):

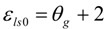
(19)

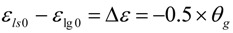
(20


**Figure 5:**
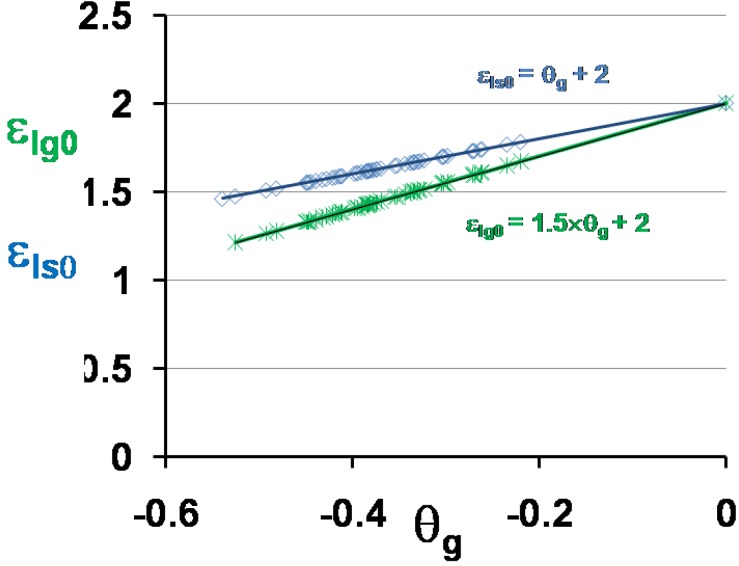
The energy saving coefficients ε_ls0_ and ε_lg0_ are calculated respectively using the scaling laws (18) and (19,20) and are plotted *versus* θ_g_ = (T_g_ − T_m_)/T_m_.

**Figure 6:**
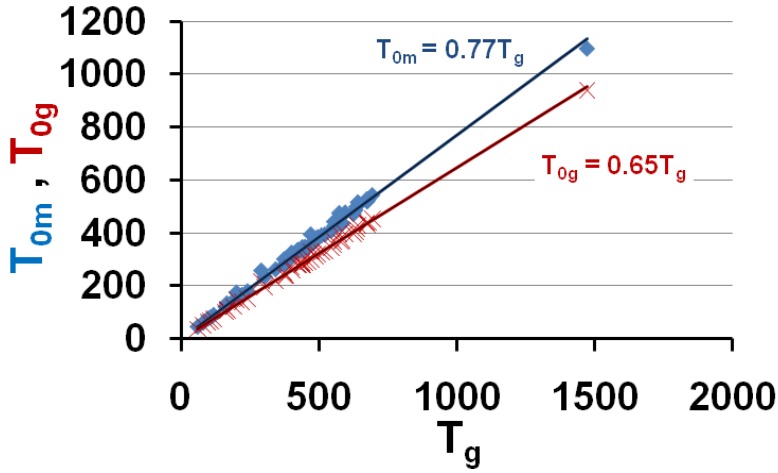
The calculated values of the free-volume disappearance temperatures T_0m_ and T_0g_ are plotted *versus* T_g_; they are equal to the VFT temperatures T_01_ and T_02_ represented in Figure 1. T_0m_ = 0.77 T_g_ and T_0g_ = 0.65 T_g_.

The two energy saving coefficients ε_ls0_ and ε_lg0_ given in Table 1 are plotted as a function of θ_g_ in Figure 5. Equations (7) and (8) are used to predict the temperatures T_0g_ and T_0m_ also given in Table 1 and plotted as a function of T_g_ in Figure 6. These values are the free-volume disappearance temperatures of glass-forming melts having a thermodynamic glass transition occurring at T*_g_ = T_g_.

**Figure 7:**
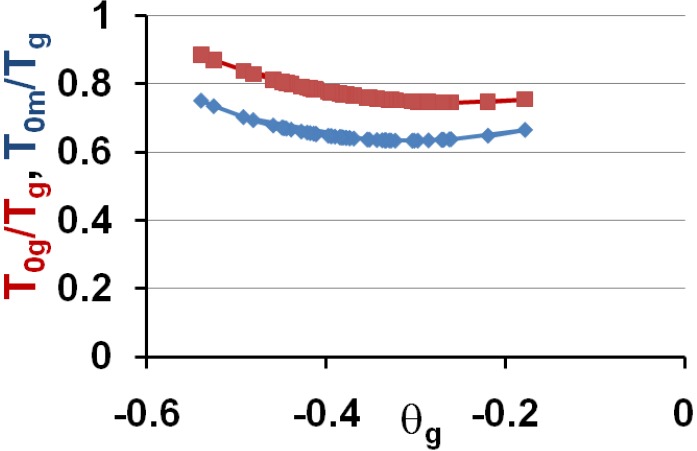
The ratios T_og_/T_g_ and T_0m_/T_g_ of the free-volume disappearance temperatures to the glass transition temperature T_g_ are plotted *versus* θ_g_ = (T_g_ − T_m_)/T_m_.

**Figure 8:**
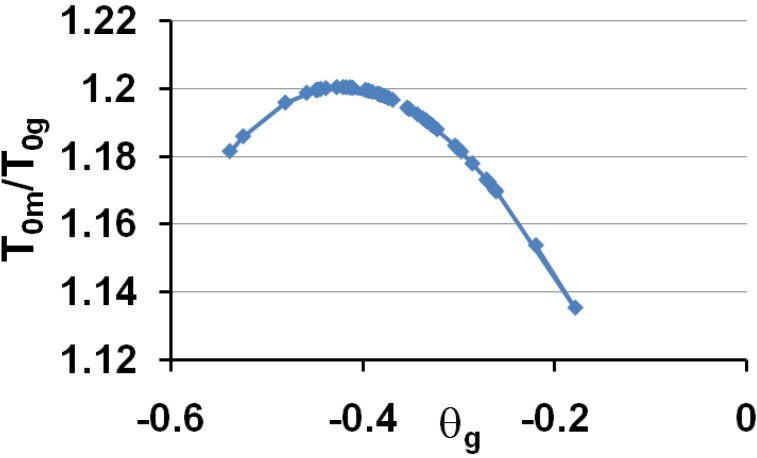
The ratios of the free-volume disappearance temperatures T_om_/T_og_ are plotted *versus* θ_g_ = (T_g_ − T_m_)/T_m_.

These predictions are in very good agreement with experiments when we compare Figure 6 to Figure 1 and Figure 4. Then, the vitreous transition corresponds to a crystal homogeneous nucleation temperature. A distribution of homogeneously-nucleated clusters is created when the temperature decreases down to T*_g_. The scaling laws (18) and (19) are obeyed and reflect intrinsic properties of glass-forming melts. The two VFT temperatures corresponding to two free-volume disappearance temperatures follow intrinsic scaling laws related to a change of the energy saving in all melts. These predictions can be more precise as shown in Figure 7 and Figure 8. In fact, the ratios T_om_/T_g_ and T_0g_/T_g_ are weakly varying with the glass transition; the proportionality coefficients 0.77 and 0.65 are mean values for a lot of glasses and polymers having θ_g_ values larger than −0.45 and smaller than −0.2. The ratio T_0m_/T_0g_ is nearly constant in the same interval of θ_g_ values as shown in Figure 6; it tends to 1 when θ_g_ tends to 0 and −2/3.

### 3.5. Volume Energy Saving Associated with Nascent Crystals in Non-Metallic Glass-Forming Melts

Volume energy saving associated with homogeneously-nucleated crystal formation also exists in non-metallic liquids. It could be due to an electrostatic interaction between a screen of ionic charges present in the melt and charges carried by homogeneously-nucleated crystals containing unoccupied ionic sites. Homogeneous nucleation gives rise, in a first step, to ultra-fine crystals among density fluctuations. Ions of opposite charges could be randomly distributed inside their own sub-lattices in a crystal. The mean charge carried by such crystals would be proportional to the square root of their atom number n. Counter-ions of opposite charge would screen the grain charge and induce an attractive interaction proportional to the square of the grain charge and then to the atom number n. Neel already made a similar assumption to explain the superparamagnetic (ferrimagnetic) properties of antiferromagnetic ultra-fine grains. The superparamagnetic Curie constant of ultra-fine grains is equal to the Curie constant of n paramagnetic atoms because the magnetic moments carried by different ions are randomly distributed in their own sub-lattices and the grain uncompensated magnetic moment is proportional to n^1/2^ [70].

The presence of volume energy saving in nonmetallic glass-forming melts is also due to a more general phenomenon associated with the formation of noncritical clusters in melts containing n atoms. The chemical potential of a small cluster is expected to differ from the bulk value. A new contribution −(p − p_0_)V_m_ ought to be added to the classical Gibbs free energy change. It depends on the Laplace pressure p applied to the cluster when a cluster is formed, p_0_ being the classical pressure of the melt on the solid particle, regardless of its size. This complementary contribution is not involved in the classical Gibbs free energy change because the pressure p is not homogeneous in the melt [71]. The Laplace pressure p increases with a decreasing atom number n. In addition, the energy saving is quantified when the critical cluster radius and the number of transferred electrons are very small in metallic glass-forming melts [14,15]. The temperature dependence of ε_ls_ given by (1) is a general law for nascent clusters in all melts.

### 3.6. Thermodynamic Origin of Relaxed Enthalpy and of Out-of-Equilibrium Nucleation Temperatures T_g_

Enthalpy is relaxed at the annealing temperature T_a_ < T*_g_ applied after quenching the undercooled melt down to a much lower temperature. The fully-relaxed enthalpy is equal to H_r_ = ΔC_plg_ (T*_g_) × [T*_g_ − T_a_] for T_k_ < T_a_ < T*_g_ instead of being related to the enthalpy excess stored by an undercooled melt quenched from T_a_ down to the Kauzmann temperature T_k_ and to the entropy available below T_a_. So this relaxed enthalpy correlated to the thermodynamic transition.

**Figure 9:**
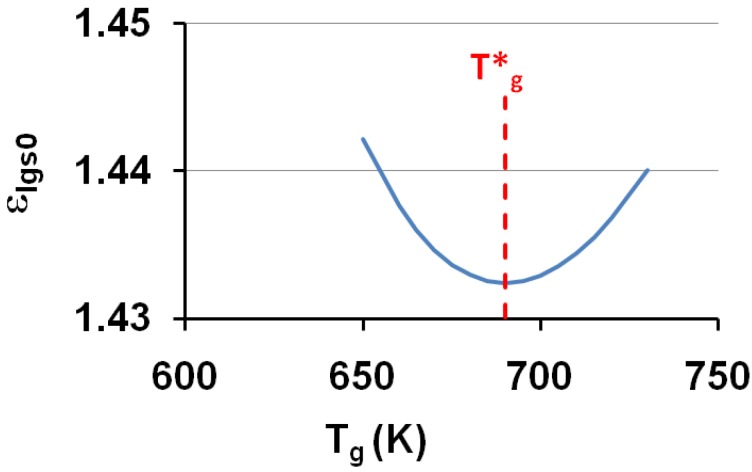
The out-of-equilibrium homogeneous nucleation temperatures T_2ls_ = T_g_ equal to homogeneous nucleation temperatures depend on energy saving coefficients ε_lgs0_ through (6). The equilibrium transition of vit 106a (Zr_58.5_Cu_15.6_Ni_12.8_Al_10.3_Nb_2.8_) at T*_g_ = 690 K has been previously determined as shown in Figure 2. The temperature T_0g_ = 437 K is equal to T_02_.

The annealing temperature T_a_ is an out-of-equilibrium homogeneous nucleation temperature of a fragile glass-forming melt and a solution of (6) corresponding, for the same value of θ_0lgs_, to an energy saving coefficient of nascent crystals at T_m_ being a little larger than the equilibrium value at a nucleation temperature equal to T*_g_ [15]. The annealing temperature T_a_ is a temporary nucleation temperature during the time lag of the transient nucleation. The undercooled melt progressively relaxes enthalpy and entropy excesses stored between T_a_ and T*_g_ towards their equilibrium values at T*_g_. The existence of this relaxed enthalpy is a strong argument in favor of a thermodynamic equilibrium at T*_g_. The time-dependent vitreous transition T_g_ is due to this endothermic heat appearing at a temperature varying with the heating rate in a DSC run. A nucleation temperature T_2ls_ = T_g_ could also exist above T*_g_ when high heating rates are used because the homogeneous nucleation temperature T_2ls_ only depends on the energy saving coefficient ε_lgs0_ for a well-defined ideal glass transition temperature T_0g_ (or θ_0g_) as shown by (6) and Figure 9.

A spin-glass transition is also characterized in zero field by the presence at once of a time-dependent susceptibility cusp temperature, a phase transition temperature and, in the magnetic field, by two lines of transition H_c_(T) and H_m_(T) [72]. The phase diagram (H,T) of Cu–Mn has been investigated by measuring magnetocaloric effects showing the importance of the entropy S(T,H) in understanding the physics of spin glass transition. The H_c_(T) is the boundary line spin-glass/non-Curie paramagnet. The H_m_(T) is a cross-over line Curie/Non-Curie paramagnet corresponding to an irreversibility line and to a freezing of rigid clusters of spins [72,73,74]. The spin-glass phase transition can be separated from the irreversibility line when the magnetic field frequency increases. The existence of this type of phenomenon in glasses remains an open question because the reversible specific heat jump always occurs in As_2_Se_3_ at the same vitreous transition regardless of the heating and cooling rates [67].

## 4. Summary and Complementary Information on the Two Crystal Nucleation Temperatures

New equations governing the crystal nucleation, reflecting the energy saving associated with Fermi energies equalization of nascent crystals and melt, have been used and applied to all glass-forming melts. The vitreous transition is characterized by freezing at a crystal homogeneous nucleation temperature only determined by thermodynamics considerations. We have shown, for the first time, that an energy scale governs the vitreous transition. This material constant does not strictly depend on the viscosity, even if the viscosity is high and nearly the same at T*_g_, because the energy barrier for crystal growth nucleation ΔG*_2ls_ divided by k_B_T_g_ is nearly the same in all glass-forming melts. The energy barrier Δf* to transfer an atom from the melt to a nascent crystal divided by k_B_T*_g_ is also nearly the same and is a little smaller than the one from transport across the melt-crystal interfaces at the first crystallization temperature which is induced by surviving intrinsic crystals.

**Figure 10:**
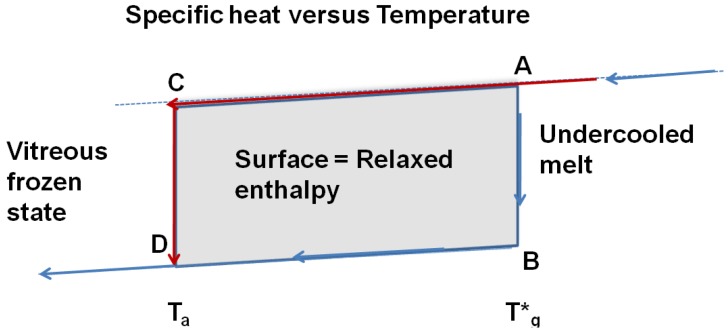
The reversible thermodynamic vitreous transition occurs at T = T*_g_; the specific heat decreases along AB; the specific heat of the vitreous fully-frozen state along BD is equal to the crystallized state. The undercooled melt is quenched at low temperatures and annealed at the temperature T_a_. The transformation from C to D relaxes an enthalpy equal to the surface ABDC when the time-lag necessary for cluster formation has evolved.

These findings are in agreement with published works having shown that the reversible specific heat jump at T*_g_ does not depend on time and on sample thermal history. In addition, the relaxed enthalpy disappears at the thermodynamic transition T*_g_ and its maximum value obtained at each annealing temperature T_a_ after quenching the undercooled liquid to lower temperatures, is given by 
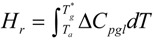
, ΔC_pgl_ being the specific heat jump at T*_g_. The apparent specific heat jump at T_g_ calculated from the heat flux measurement is equal to the reversible one. The specific heat jump deduced from heat flux measurement occurs at a temperature T_g_ depending on the heating rate. There is no visible anomaly at T*_g_ in a DSC run. This phenomenon is schematized in Figure 10. The glass transition temperatures T_g_ determined by DSC correspond to out-of-equilibrium crystal homogeneous nucleation temperatures and to out-of-equilibrium values of the energy saving coefficient ε_lg0_.

The thermodynamic transition T*_g_ is a linear function of the energy saving divided by the fusion heat associated, in a nascent crystal formation, with the equalization of Fermi energy or chemical potential of nascent crystals and glass-forming melts. It is possible to predict, only using T*_g_ and the melting temperature T_m_, a free-volume disappearance temperature equal to the VFT temperature of fragile-glass-forming melts deduced from viscosity and relaxation time measurements above and near T*_g_. There are two crystal homogeneous nucleation temperatures which follow scaling laws linearly dependent on two energy saving coefficients ε_ls0_ in the crystal formation as there are two VFT temperatures.

Experimentally, the first-crystallization temperature occurs when cooling the glass-forming melt at a lesser rate than the critical one, down to a temperature that is higher than the homogeneous nucleation temperature and is generally induced by intrinsic heterogeneous crystals which reduce the energy barrier for crystal growth. The isothermal crystallization time depends on overheating and undercooling temperatures and leads to a time-temperature-transformation diagram induced by intrinsic nuclei. The nose temperature of this diagram depends on the overheating temperature, the surviving crystal size and the energy saving ε_ls_.

The second nucleation temperature is lower and gives rise by homogeneous nucleation to imperfect crystals having an energy barrier for diffusion from the melt to the crystal slightly smaller than the first. The free-volume disappearance temperature of the undercooled melt decreases from T_0m_ to T_0g_. A glass state is obtained by quenching the melt using a cooling rate larger than its critical value. The vitreous transition temperature T*_g_ occurs at a homogeneous nucleation maximum-rate temperature determined by a smaller value ε_lgs_(θ_g_) of the energy saving associated with a smaller VFT temperature. The relaxation time leading to vitreous state is the time lag for initial formation of a homogeneously-nucleated-cluster distribution during the transient nucleation. These entities could be imperfect crystals. Their formation is a preliminary step during the long time leading to crystallization. The time dependence of various properties depends on the time lag τ^ns^ associated to the transient nucleation and to the steady-state nucleation time t_sn_ depending on the energy barrier for crystal growth.

The model used in this paper is also based on previous publications related to the classical Gibbs free energy change for a crystal formation in an undercooled melt that has been completed by an energy saving associated with the equalization of Fermi energies or chemical potentials of melt and nascent crystal. This analysis only works for nascent crystals in an out-of-equilibrium state having a radius smaller than the critical radius for crystal growth because J. W. Gibbs’s phase coexistence rule predicts the absence of energy saving for radii larger than the critical one when solid and liquid phases are at equilibrium at the melting temperature.

## 5. Conclusions

The vitreous transition is a new type of phase transition from undercooled melt to frozen state, without entropy and enthalpy change occurring at a temperature T*_g_, which corresponds to the maximum nucleation rate temperature of homogeneously-nucleated crystals in bulk metallic and non-metallic glass-forming melts. Because of the melt freezing, the steady-state nucleation time is too long to ever reach the divergence of the correlation length in critical phenomenon and the crystal’s growth.

These nascent crystals would be formed with a free energy change which differs from the classical Gibbs free energy change used in many nucleation models. A complementary energy saving exists which depends on the atom or molecule number n involved in these crystals. We have shown the existence of two homogeneous nucleation temperatures associated with two energy savings, which follow scaling laws as a function of the vitreous transition temperature. The nascent crystals acting at the vitreous transition could contain a lot of unoccupied ionic sites as compared with crystals surviving in the melt and acting as growth nuclei at higher temperatures.

The glass freezing occurs without entropy and enthalpy changes; these changes can only appear at unattainable times when crystallization occurs. This analysis shows that the frozen and the solid states have the same equilibrium specific heat below the glass transition, eliminating all speculations about other configurational contributions and phase transitions.

The disappearance temperature of the fully-relaxed enthalpy, as described in previous publications, does not depend on time and is equal to the thermodynamic temperature T*g. The specific heat jump accompanying this phase transition is deduced from the linear variation of the relaxed enthalpy with temperature. The DSC runs are not able to detect T*_g_ because the enthalpy is continuous at this temperature.

The existence of a vitreous transition viewed as a constant of material was initially established by experiments eight years ago and published by the University of Pardubice.
